# The Lamin Proteins in Nuclear Structure, Functions, and Laminopathies

**DOI:** 10.3390/cells15121051

**Published:** 2026-06-08

**Authors:** Gan Zhao, Ziheng Chen, Caifeng Yang, Mingzheng Liu, Weiyong Wang, Chuanmao Zhang

**Affiliations:** 1 The Academy for Cell and Life Health, Faculty of Life Science and Technology, Kunming University of Science and Technology, Kunming 650500, China; zhaog@kust.edu.cn (G.Z.);; 2 The Ministry of Education Key Laboratory of Cell Proliferation and Differentiation, College of Life Sciences, Peking University, Beijing 100871, China

**Keywords:** lamins, laminopathies, nuclear lamina, genome organization, progeroid syndromes

## Abstract

The lamin proteins are classified into A- and B-types, and together with their associated proteins, they form the nuclear lamina, which governs diverse nuclear structures and functions, including nuclear mechanics, chromatin organization, and gene regulation. Mutations of these proteins give rise to a strikingly diverse group of tissue-specific disorders, the laminopathies, including muscular dystrophies, cardiomyopathies, lipodystrophies, neuropathies, and premature aging syndromes, despite their broad expression. Unraveling the basis of this tissue selectivity has revealed that lamins function not merely as structural elements but as active regulators. While the A-type lamins modulate nuclear stiffness, transcription, and genome integrity, the B-type lamins ensure mechanical resilience and heterochromatin tethering. Pathogenic mutations of these proteins disrupt their functions through convergent mechanisms that manifest according to tissue-specific contexts, leading to impaired nuclear mechanics, aberrant gene regulation, defective DNA repair, and cellular senescence. Advances in patient-derived cellular models and animal systems have illuminated these vulnerabilities and catalyzed therapeutic progress, ranging from farnesyltransferase inhibitors to emerging genome-editing strategies. Collectively, studies of lamin protein function reveal how the nucleus maintains its structures and functions, while studies of laminopathies demonstrate how nuclear dysfunction drives systemic disease and points toward mechanism-based therapies.

## 1. Introduction

The nuclear envelope in higher eukaryotic cells consists of an outer nuclear membrane (ONM), an inner nuclear membrane (INM), and many nuclear pore complexes (NPCs) sitting at the fusion sites of both membranes to form material transportation channels between the nucleoplasm and the cytoplasm [1,2]. Beneath the INM lies the nuclear lamina, a filamentous protein meshwork that serves as the principal skeletal framework of the nucleus. As such, it is essential for maintaining nuclear integrity, organizing higher-order chromatin architecture, regulating gene expression programs, and transmitting mechanical signals between the cytoskeleton and the genome [3]. Notably, these functions place the nuclear lamina at the intersection of nuclear mechanics, genome regulation, and cellular signaling.

The nuclear lamina is primarily composed of a family of type V intermediate filament proteins, the lamins, and their associated proteins. The lamin proteins are classified into the A-type and the B-type, and while the A-type lamins, including both lamins A and C, are generated by alternative splicing of the *LMNA* gene, the B-type lamins, containing both lamins B1 and B2, are encoded by *LMNB1* and *LMNB2* genes, respectively [4]. The A-type lamins are developmentally regulated and are predominantly expressed in differentiated tissues, whereas the B-type lamins are constitutively expressed in nearly all nucleated cells [5,6]. Extensive protein–protein and protein–chromatin interactions determine nuclear shape, size, and mechanical properties, and coordinate fundamental nuclear functions, including transcriptional regulation, DNA damage responses, and cell cycle control [7,8,9,10,11].

Structural or functional disruption of lamins and their associated proteins results in a group of severe inherited disorders, collectively termed laminopathies [12,13]. The clinical spectrum of laminopathies is remarkably broad, encompassing striated muscle diseases (such as Emery–Dreifuss muscular dystrophy, EDMD, and Dilated cardiomyopathy, DCM), lipodystrophies (such as Dunnigan-type familial partial lipodystrophy, FPLD2), peripheral neuropathies, and segmental progeroid syndromes that accelerate multiple aspects of aging, most notably Hutchinson-Gilford progeria syndrome (HGPS) [14,15]. However, despite extensive investigations, the precise pathogenic mechanisms underlying their tissue specificity remain incompletely understood, suggesting that multiple, partially overlapping mechanisms may contribute to the development of these diseases or disorders.

Here, we summarize recent advances in understanding lamins as multifunctional nuclear proteins and examine how their structural properties, isoform-specific functions, and interaction networks contribute to cellular homeostasis. We also discuss the molecular mechanisms underlying the major laminopathy classes, highlighting both the shared and disease-specific pathogenic pathways. 

## 2. The Nuclear Lamins

### 2.1. Composition Classification and Biogenesis of Lamins

The vertebrate nuclear lamina is a proteinaceous network underlying the INM, composed primarily of lamin proteins encoded by three principal genes: *LMNA*, *LMNB1*, and *LMNB2*. Phylogenetic analyses indicate that lamins represent the evolutionary ancestors of the entire intermediate filament superfamily [4]. Although initially thought to be restricted to metazoans, lamin-like proteins have since been identified in diverse unicellular eukaryotes, including the amoebozoan *Dictyostelium discoideum*, suggesting that the origin of lamin can be traced back to the last eukaryotic common ancestor [16,17,18]. The diversification into A- and B-type lamins represents a defining feature of vertebrate evolution, although the selective pressures driving this specialization remain incompletely understood.

Phylogenetic analyses indicate that lamins represent the evolutionary ancestors of the entire intermediate filament superfamily, emerging prior to the divergence of metazoans. Diversification into A- and B-type lamins represents a defining feature of vertebrate evolution, although the selective pressures driving this specialization remain incompletely understood.

**Figure 1:**
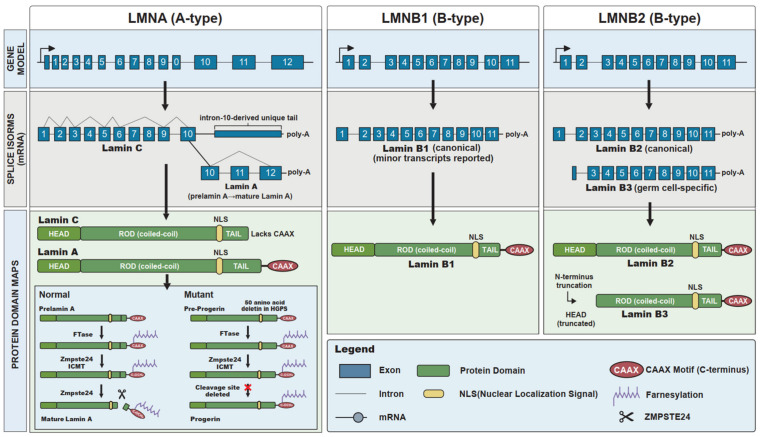
The structure and alternative splicing of A-type and B-type lamins. All lamin proteins share a conserved tripartite domain structure consisting of an N-terminal head, a central rod domain, and a C-terminal tail carrying a nuclear localization signal. Alternative splicing of *LMNA* produces lamin A and lamin C. Lamin C has a C-terminal tail encoded by intron 10 that lacks the CAAX motif. In contrast, lamin A is produced from prelamin A through multiple processing steps. The mutant prelamin A lacking 50 amino acids loses the cleavage site for the key processing enzyme ZMPSTE24, thereby generating the progerin. B-type lamins are translated more simply and retain the CAAX motif. Lamin B1 derives from *LMNB1*, while lamin B2 and lamin B3 derive from *LMNB2*. Lamin B3 is a germ cell-specific truncated isoform lacking part of its N-terminal region.

B-type lamins—lamin B1 and lamin B2—are encoded by the *LMNB1* and *LMNB2* genes, which are located on human chromosomes 5q23.2 and 19p13.3, respectively. Each gene typically gives rise to a single major protein product. B-type lamins are constitutively expressed in nearly all nucleated cells from the earliest stages of embryogenesis, as they are indispensable for cellular viability [3,6]. Both lamin B1 and lamin B2 harbor a C-terminal CAAX motif that undergoes permanent farnesylation, anchoring these proteins to the INM (Figure 1). At the subcellular level, B-type lamin proteins are highly enriched at the nuclear periphery, where they form a stable filamentous meshwork closely associated with NPCs and membrane-embedded nuclear lamina-associated proteins. Through homotypic and heterotypic interactions, B-type lamins polymerize into higher-order filaments that provide a persistent scaffold for nuclear envelope organization throughout the cell cycle. Some vertebrates express lamin B3, a meiosis-associated B-type lamin detected primarily in germ cells; although less extensively characterized, its presence suggests additional structural diversification in specialized cellular contexts.

A-type lamins include lamin A, lamin C, and the germline-specific isoform lamin C2, all derived from the *LMNA* locus through alternative splicing. Lamin A (664 amino acids) and lamin C (572 amino acids) share an identical N-terminal head and rod domain but diverge in their C-terminal tails. Lamin C lacks the C-terminal CAAX motif and is therefore not prenylated. In contrast, lamin A is synthesized as a precursor protein, prelamin A, that undergoes a series of post-translational processing. In contrast to B-type lamins, A-type lamins display more dynamic subcellular distribution. In addition to incorporation into the peripheral nuclear lamina, a substantial fraction localizes within the nucleoplasm, forming a more soluble and mobile pool [19]. This dual localization reflects distinct assembly properties and interaction networks and is sensitive to cellular differentiation state and mechanical context. The *LMNA* locus is a major hotspot for human laminopathies, with hundreds of pathogenic variants identified across the coding region. Notably, these mutations predominantly disrupt lamin structure, assembly, or post-translational processing, emphasizing the central importance of protein integrity rather than transcriptional dosage in disease pathogenesis [20].

The biogenesis of mature lamin A involves a tightly regulated, multi-step post-translational maturation pathway. Prelamin A is initially farnesylated at its C-terminal CAAX motif, followed by endoproteolytic removal of the terminal tripeptide and carboxyl methylation. A second cleavage event, catalyzed by the zinc metalloprotease *ZMPSTE24*, removes the final 15 amino acids, including the farnesylated cysteine, yielding mature, non-farnesylated lamin A [21,22]. The failure of this processing cascade, attributable either to *LMNA* mutations that disrupt the *ZMPSTE24* cleavage site or to inherent defects within *ZMPSTE24* itself, leads to accumulation of permanently farnesylated prelamin A or its truncated form, progerin, which underlies several progeroid disorders.

At the molecular level, lamins self-assemble through a hierarchical process. Individual lamin monomers first associate in parallel to form coiled-coil dimers via their central rod domains. These dimers subsequently align head-to-tail to generate polar protofilaments, which associate laterally into higher-order filaments beneath the INM. Direct lamin–lamin interactions contribute to nuclear lamina assembly, although B-type lamins are generally considered to establish a more stable membrane-proximal scaffold onto which A-type lamins are incorporated [23]. The immunoglobulin-like fold within the C-terminal tail domain plays a critical role in mediating intermolecular contacts and interactions with lamin-binding partners, including nuclear envelope transmembrane proteins and chromatin-associated factors [24].

As a result, the nuclear lamina is not a uniform entity but a composite and dynamic network whose composition, thickness, and biophysical properties vary across cell types and developmental stages. A key corollary is that this structural plasticity likely underlies the capacity of the nuclear lamina to support diverse nuclear functions discussed below.

### 2.2. Multifaceted Functions of Lamins

#### 2.2.1. Lamins as the Skeleton of the Nuclear Envelope

**Figure 2:**
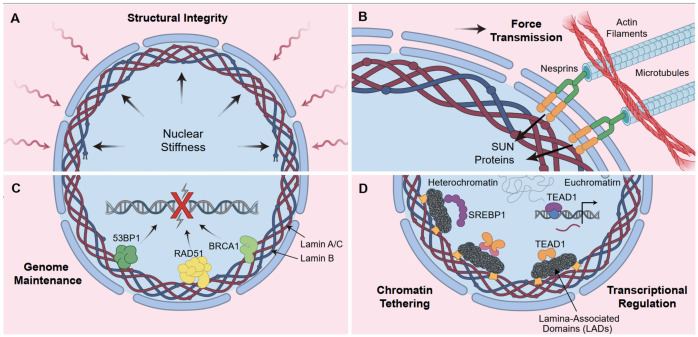
The role of lamins in nuclear structure and function. (**A**) Lamin A/C and lamin B together define nuclear stiffness and elasticity, maintaining nuclear shape and preserving structural integrity under mechanical stress. (**B**) Through coupling with the linker of nucleoskeleton and cytoskeleton complex (LINC), lamin proteins transmit cytoskeletal forces generated by actin filaments and microtubules into the nucleus, enabling mechanosensitive transcriptional responses. (**C**) The nuclear lamina also participates in genome maintenance by creating specialized microenvironments for DNA damage response factors near the nuclear periphery. (**D**) At the nuclear periphery, lamins tether lamin-associated domains (LADs) and organize higher-order chromatin architecture, contributing to transcriptional regulation.

One fundamental function of the nuclear lamina is to serve as the primary load-bearing scaffold of the nucleus, thereby determining mechanical integrity and resistance to deformation (Figure 2A). Lamin filaments exhibit viscoelastic behavior arising from their polymeric assembly into a dense, cross-linked network beneath the INM [25]. Unlike actin filaments or microtubules, lamin proteins form a continuous peripheral shell that distributes mechanical stress across the nuclear surface.

At the molecular level, the skeletal function of lamins derives from their ability to form long-lived filamentous assemblies that are mechanically coupled to both chromatin and the cytoskeleton. Through direct interactions with INM proteins and the linker of nucleoskeleton and cytoskeleton complex (LINC), lamin networks transmit forces from the cytoskeleton to the nuclear interior, effectively integrating the nucleus into the cellular mechanical continuum.

Biomechanical studies demonstrate a functional division of labor between lamin isoforms: A-type lamins primarily contribute to nuclear stiffness and viscosity, whereas B-type lamins maintain elasticity and stabilize LINCs at the nuclear envelope [25,26,27,28]. Notably, tissues exposed to high mechanical stress, such as cardiac and skeletal muscle, express elevated levels of lamin A/C, reinforcing nuclear integrity under load.

Disruption of nuclear lamina assembly, particularly through pathogenic alterations affecting A-type lamins, compromises nuclear mechanics and increases susceptibility to nuclear rupture under stress, forming the basis of the structural hypothesis of laminopathies [7,23]. Dysregulated Lamin A/C phosphorylation compromises nuclear envelope integrity [29]. Chromatin is also involved in nuclear mechanical properties, and this involvement is deformation-dependent [30]. Thus, mechanotransduction and chromatin regulation are highly interdependent, with the relative contributions of nuclear lamina defects likely varying across different tissue contexts and mechanical environments [31]. Collectively, while the structural hypothesis remains a valuable foundation, a more comprehensive understanding of laminopathies requires considering the interplay between nuclear mechanics, chromatin organization, and tissue-specific factors.

#### 2.2.2. Lamins in Mechanical Signaling and Force Transmission

Mechanical signaling, the process by which cells convert mechanical stimuli into biochemical signals, depends critically on the physical linkage provided by the LINC (Figure 2B). This complex spans the nuclear envelope, linking the cytoskeleton via ONM nesprins to the nucleoskeleton through INM SUN proteins and lamins [7]. Through this coupling, forces generated at the cell surface or within the cytoskeleton are transmitted directly to the nuclear interior. The nucleus itself functions as a mechanosensitive organelle. Nuclear deformation can alter chromatin organization and modulate mechanosensitive transcription factors, thereby influencing gene expression programs involved in differentiation, migration, and tissue homeostasis [7,32]. For instance, studies demonstrated that the TEA domain transcription factor 1 (TEAD1), a downstream effector of the Hippo pathway, can be aberrantly sequestered at the nuclear periphery by mutant lamin A/C Q353R, resulting in impaired transcriptional activation and contributing to DCM [33].

The pathological consequences of defective nuclear mechanics are evident in several laminopathies. In EDMD and *LMNA* mutation-related DCM, compromised nuclear lamina integrity fails to protect nuclei from repetitive mechanical stress generated during muscle contraction, leading to nuclear envelope rupture, DNA damage, and progressive cell loss [23,34]. Furthermore, studies reported that muscle-specific nuclear envelope proteins such as Net39 are required to protect nuclei from mechanical stretch, and their loss phenocopied aspects of EDMD pathology [35]. Similarly, work in *Drosophila* germline stem cells showed that emerin is required to maintain nuclear lamina organization during semi-closed mitosis under mechanical constraint, linking nuclear lamina integrity to stem cell maintenance [36]. During cell migration, the nucleus often constitutes a rate-limiting physical barrier. In HGPS, accumulation of progerin increases nuclear stiffness and restricts nuclear deformability, impairing migration through confined three-dimensional environments [8]. In contrast, increased nuclear deformability, for example, following *LMNA* depletion, can enhance DNA break mobility under certain conditions, highlighting a complex relationship between nuclear mechanics and genome stability [26,37].

Thus, the structural role of the nuclear lamina is closely integrated with mechanical signaling. Mutations that disrupt this system not only weaken nuclear mechanics but also alter mechanical feedback pathways required for tissue maintenance. This relationship may partly explain the selective vulnerability of mechanically stressed tissues in many laminopathies.

#### 2.2.3. Lamins in Genome Stability and DNA Repair

A further critical function of the nuclear lamina is the preservation of genome stability through spatial organization of DNA damage responses and modulation of DNA repair pathway choice (Figure 2C). Beyond acting as a passive structural barrier, the nuclear lamina provides a scaffold that contributes to the recruitment and spatial coordination of repair factors.

Key DNA damage response proteins, including p53-binding protein 1 (53BP1) and breast cancer type 1 susceptibility protein (BRCA1), localize to the nuclear periphery in a lamin-dependent manner, suggesting that lamins contribute to the formation of repair microenvironments [11]. In undamaged cells, 53BP1 directly binds lamin A/C as a nucleoskeleton protein, but this interaction is abrogated upon DNA damage. Nevertheless, lamin A/C continues to regulate both the steady-state levels and the nucleoplasmic pool of 53BP1, enabling its efficient recruitment to damage sites; consequently, lamin A/C-null cells exhibit impaired 53BP1 recruitment and compromised DNA repair [38]. Notably, 53BP1 also preserves heterochromatin integrity through liquid–liquid phase separation with HP1α, an activity that is genetically separable from its established role in double-strand break (DSB) repair [39]. Emerging evidence further indicates that A-type lamins maintain the levels of key DNA repair proteins, including 53BP1, BRCA1, and RAD51, thereby promoting both non-homologous end joining (NHEJ) and homologous recombination [40]. Collectively, these findings establish that the nuclear lamina actively couples nuclear architecture with DNA repair fidelity and genome stability by organizing both the physical mobility of broken DNA and the spatial availability of core repair factors.

Under oxidative stress, lamin C undergoes phase separation to form gel-like nuclear condensates, which sequester and protect key DNA replication factors, such as PCNA and POLD1 [41]. This spatial compartmentalization prevents the damage or dispersion of replication machinery during stress, and upon stress removal, the condensates disassemble to allow timely recovery of DNA replication. Thus, lamin C condensates act as dynamic, redox-responsive storage hubs that safeguard replication competence, revealing a novel paradigm of how the nuclear lamina contributes to genome stability beyond its classical structural roles.

Recent studies have identified specialized nucleoskeleton assemblies linking lamins to DSB repair. The proteins IFFO1 and IFFO2 form filamentous networks with lamin A/C and interact with the NHEJ factor XRCC4, generating a scaffold that promotes efficient NHEJ while limiting aberrant chromosomal rearrangements [42]. This structure appears to functionally complement XRCC4-like factor and contributes to repair efficiency and fidelity.

Mutations in lamin proteins, particularly those associated with progeroid syndromes, disrupt genome maintenance. A prominent consequence is the accumulation of persistent DNA damage and genomic instability, features commonly observed in both pathological and physiological aging [43]. Cells derived from patients with HGPS exhibit pronounced DNA repair defects, and progerin has been identified as a major contributor to this dysfunction [44]. Mechanistically, alterations in lamin A/C can influence DSB repair pathway choice. While homologous recombination predominates in S/G2 phases, NHEJ is more prevalent in G0/G1. Evidence suggests that accumulation of progerin or prelamin A shifts repair toward NHEJ at the expense of homologous recombination. Moreover, within homologous recombination, mutant lamins appear to favor gene conversion over crossover events, potentially increasing the risk of loss of heterozygosity [11]. This shift in pathway utilization likely contributes to elevated mutational burden.

The relationship between lamin proteins and genome stability further extends to DNA replication and telomere biology. Lamins contribute to stalled-replication-fork protection, and their dysfunction can induce replication stress and deplete deoxynucleotide triphosphate (dNTP) pools [11]. Telomeres are also functionally connected to the nuclear envelope. Progressive telomere shortening in normal human fibroblasts was reported to activate the same cryptic splice site in *LMNA* that operates constitutively in HGPS, resulting in low-level progerin production [45]. This progerin accumulation may promote further cellular senescence, establishing a feedback loop linking telomere attrition and nuclear aging [44,46]. This mechanism may partly explain the presence of progerin in normally aging cells, positioning lamin A as a potential integrator of multiple aging-associated stress signals.

Collectively, these findings establish a key emerging concept that the mechanical integrity of the nuclear lamina is not merely a passive structural support but an active determinant of DNA repair capacity and genome stability, with broad implications for laminopathies, cancer biology and aging.

#### 2.2.4. Lamins in Genome Organization and Transcriptional Regulation

Another essential function of the nuclear lamina is the organization of three-dimensional genome architecture and the regulation of transcriptional programs (Figure 2D). This function is mainly achieved by anchoring genomic regions to the nuclear periphery, thereby forming lamina-associated domains (LADs). LADs are large, heterochromatic regions characterized by low gene density, repressive histone marks, and transcriptional activity [9,47].

Lamins interact with chromatin through both direct and indirect mechanisms. Structural studies identified a conserved motif within the lamin A tail domain that binds the acidic patch of the H2A–H2B histone dimer, providing a molecular basis for nucleosome interaction [48]. In addition, lamin proteins establish an indirect but critical connection to chromatin through integral INM proteins harboring the LEM domain, including LAP2, emerin, and MAN1, which act as molecular adaptors [49]. This interaction is reinforced by adaptor proteins such as Barrier-to-autointegration factor (BAF)—which binds both DNA and the immunoglobulin-like fold of lamin A/C—stabilizing the nuclear lamina-chromatin interface [20,50]. BAF functions as a homodimer that simultaneously binds DNA, lamins, and LEM-domain proteins, thereby tethering peripheral heterochromatin to the nuclear lamina [6,51]. This lamin-LEM-BAF-chromatin axis is essential for maintaining nuclear architecture and heterochromatin positioning [52]. Furthermore, distinct lamin isoforms preferentially associate with different chromatin subcompartments: lamin B1 is enriched at facultative heterochromatin, whereas lamin B2 shows stronger association with constitutive heterochromatin, suggesting isoform-specific contributions to genome organization [53].

Nuclear lamina-chromatin interactions are highly dynamic during development and differentiation. In pluripotent cells, heterochromatin tethering is largely mediated by the lamin B receptor (LBR) [54]. LBR interacts with lamin, forming part of the nuclear envelope and stabilizing heterochromatin [55,56]. During mitosis, it participates in nuclear envelope reconstruction and energy metabolism, whereas in differentiated cells this role is progressively assumed by lamin A/C [57,58,59]. Furthermore, LADs themselves are heterogeneous, comprising both constitutive domains that remain peripherally anchored and facultative domains that can detach in a cell-type–specific manner to permit gene activation. Lamins are also associated with transcriptionally active regions in the nuclear interior, indicating functions beyond peripheral gene repression. Single-cell analyses further demonstrate substantial variability in LAD positioning, highlighting a stochastic dimension of nuclear lamina-mediated genome regulation [9]. In human embryonic stem cells, complete lamin depletion barely affects genome–nuclear lamina interactions; however, upon differentiation into mesenchymal stem cells, the same depletion triggers extensive chromatin repositioning, chromosome territory disruption, and nuclear speckle mislocalization [60]. This demonstrates that the dependency and functional impact of lamina–chromatin interactions shift fundamentally during differentiation, rendering these interactions highly dynamic.

Disruption of nuclear lamina-mediated chromatin organization represents a major pathogenic mechanism in laminopathies and aligns with the gene expression hypothesis. Mutations in *LMNA* can induce large-scale alterations in genome organization. For example, studies of Mandibuloacral dysplasia (MAD) reported widespread LAD repositioning, loss of chromatin compartmentalization, and shortening of topologically associating domains (TADs), leading to dysregulation of senescence-associated genes [61]. Similarly, lipodystrophy-associated mutations, such as R482W, impair lamin A/C interactions with chromatin regulators and specific genomic loci, while structural analyses identified residues, including K486 and H506, as critical for nucleosome contact [20,62].

Transcriptional dysregulation in laminopathies extends beyond chromatin repositioning to include altered regulation of transcription factors. In DCM associated with the *LMNA* Q353R mutation, mutant lamin A/C aberrantly sequesters the TEAD1 at the nuclear periphery, preventing activation of cardiac maturation genes [33]. Additional mechanisms involve dysregulation of signaling pathways controlling transcription factor activity. For instance, hyperactivation of the mitogen-activated protein kinase (MAPK)/extracellular signal-regulated kinase (ERK) pathway promotes cytoplasmic sequestration of myocardin-related transcription factor A (MRTF-A), thereby impairing serum response factor (SRF)-dependent transcription, which is essential for cardiomyocyte function [63]. Furthermore, mutant lamins can interfere with nuclear hormone signaling, as seen with the *LMNA* Q353R mutation, which impairs vitamin D receptor (VDR) function and DNA repair gene expression [64,65]. Taken together, these observations suggest that lamin mutations disrupt transcriptional programs both by altering genome organization and by perturbing regulatory machinery that interprets chromatin states, thereby contributing to tissue-specific transcriptional changes observed in laminopathies.

#### 2.2.5. Lamins During Mitosis

During mitosis, lamins undergo profound changes distinct from their interphase state, characterized primarily by a dramatic shift in their post-translational modification profile, particularly a sharp increase in phosphorylation [66]. This directly leads to fundamental alterations in their higher-order structure and subcellular localization, consequently shifting their function from supporting nuclear architecture in interphase to actively participating in nuclear envelope dynamics during mitosis [67]. During interphase, lamins and associated nuclear envelope proteins form a mechanically responsive scaffold that coordinates nuclear integrity, chromatin organization, and gene regulation [7,8,23]. However, upon entering mitosis, lamins are hyperphosphorylated by multiple kinases. During this process, B-type lamins remain continuously associated with a population of vesicles derived from the disassembled nuclear envelope. In contrast, A-type lamins become soluble and diffuse throughout the cytoplasm as small oligomers [1,68]. This differential localization reflects the distinct spatial fates of the two lamin types during mitosis.

As cells enter early mitosis, the nuclear lamina must be depolymerized, driven by hyperphosphorylation, to enable successful nuclear envelope breakdown (NEBD) [69,70]. Key mitotic kinases, most notably cyclin-dependent kinase 1, Polo-like kinase 1, and Aurora kinases, act in concert to trigger extensive phosphorylation of numerous nuclear envelope proteins, including lamins and INM proteins [71,72]. Protein kinase C (PKC) activity has also been implicated in regulating lamin A/C mobility during interphase [73]. This hyperphosphorylation culminates in disruption of protein interactions, loss of nuclear lamina integrity, and detachment of INM proteins from condensing chromosomes, thereby transitioning the lamina meshwork from a solid-state to a soluble, dispersed form Ref. [74]. Consequently, at this stage, lamins undergo a functional transformation from stable structural scaffolds into highly dynamic complexes associated with membranes or cytoplasmic components.

At mitotic exit, dephosphorylation of lamins promotes repolymerization and drives nuclear envelope reassembly around segregated chromosomes. Protein phosphatases, particularly PP1 and PP2A, are activated upon mitotic exit and precisely and sequentially remove the phosphate groups added by mitotic kinases to the lamins [75]. B-type lamins are recruited early in telophase to condense chromosome surfaces and rapidly enclose the decondensing chromatin, followed by the delayed incorporation of A-type lamins after nuclear pore assembly, thereby restoring nuclear architecture and mechanical integrity in daughter cells [76]. As dephosphorylation proceeds, lamins and other nuclear envelope proteins limit aberrant chromatin exposure by gradually forming a new lamina meshwork around reforming daughter nuclei, thereby re-establishing nuclear envelope integrity.

Collectively, lamin proteins play two distinct and opposing roles during mitosis entry and exit. When entering mitosis, lamins undergo kinase-mediated hyperphosphorylation, leading to NEBD. Upon exiting mitosis, dephosphorylation is reactivated, allowing lamins to regain their polymerization capacity and act as directors to orchestrate the precise assembly of the new nuclear envelope. This cycle is a fundamental mechanism ensuring the correct segregation of genetic material and the orderly progression of the cell cycle.

## 3. Laminopathies and Their Clinical Manifestations

Laminopathies represent a clinically diverse class of human diseases caused by mutations in genes encoding components of the nuclear lamina and associated nuclear envelope proteins. Here, we summarize the mutation sites, phenotypes, and underlying molecular mechanisms of laminopathies (Table 1). Although mutations in *LMNA* account for the majority of reported cases, disease-causing alterations in B-type lamins, particularly *LMNB1*, as well as mutations in other nuclear envelope proteins, also give rise to distinct laminopathy phenotypes [13,77].

The pathogenesis of laminopathies is explained through several interconnected mechanistic frameworks. The classical structural hypothesis attributes disease to compromised nuclear integrity and impaired mechanical signaling, leading to stress-induced cellular damage, particularly in striated muscle tissues [23]. In contrast, the “gene expression hypothesis” emphasizes that lamin mutations disrupt chromatin organization and intracellular signaling pathways, thereby altering transcriptional programs [12,119]. More recently, these perspectives have been integrated with models highlighting cellular senescence, stem cell exhaustion, and chronic inflammation as additional pathogenic contributors, especially in progeroid syndromes [112]. Current evidence suggests that these mechanisms are not mutually exclusive but operate within an interconnected and synergistic network that drives disease progression [120].

In laminopathies, mutations affect structural components present in nearly all nucleated cells. Nevertheless, laminopathies exhibit marked tissue-specific vulnerability, predominantly affecting striated muscle, adipose tissue, peripheral nerves, or, in some cases, causing systemic premature aging [12,15]. This tissue selectivity likely arises from the interaction between a specific lamin mutation and the distinct mechanical demands, transcriptional programs, and developmental context of individual tissues [15,119]. Consequently, although laminopathies share common molecular roots, their clinical manifestations are highly system-oriented.

For this reason, laminopathies are conventionally classified according to the primary tissue or organ system affected, despite substantial phenotypic overlap among categories [14]. This classification provides a clinically practical framework while acknowledging that shared pathogenic mechanisms underlie seemingly distinct disease entities.

Striated muscle laminopathies represent a major disease category. Mutations in *LMNA* are a leading cause of familial DCM with conduction system disease, characterized by progressive heart failure and an increased risk of malignant arrhythmias [121,122,123]. Mutations in emerin or lamin A/C can lead to EDMD, which presents with early contractures, slowly progressive muscle weakness, and cardiac involvement [23]. Emerin is retained in the INM through direct interaction with lamin A/C. The two different regions of lamin A/C are sufficient to correctly locate emerin in the INM and prevent lateral diffusion of emerin within the INM. Stable anchoring of emerin to the INM requires assembling A-type lamins into a filamentous network [124]. The pathogenic mechanisms in muscle tissue frequently align with the structural hypothesis. The mechanically stressed environment of contracting muscle imposes substantial strain on the nucleus. A compromised nuclear lamina resulting from *LMNA* or *EMD* mutations promotes nuclear fragility, recurrent nuclear envelope rupture, and DNA damage accumulation, ultimately triggering cell death and tissue degeneration [23,34,96]. However, mechanical failure alone does not fully account for disease progression. Altered signaling pathways also contribute significantly. Studies demonstrate that many *LMNA*-associated cardiomyopathy mutations lead to constitutive activation of the MAPK/ERK pathway, promoting pathological remodeling and apoptosis in cardiomyocytes [90]. Furthermore, disrupted nucleo–cytoskeletal coupling through the LINC may impair cytoskeletal gene expression and contribute to sarcomere disorganization [63]. Emerging evidence also suggests that secondary organelle stress, including Golgi fragmentation and activation of the unfolded protein response, may further exacerbate disease pathogenesis [34].

Adipose tissue laminopathies, most notably FPLD2, are caused by specific heterozygous missense mutations in *LMNA* (e.g., R482W/Q) [100,101,102]. Affected individuals exhibit progressive loss of subcutaneous adipose tissue from the extremities and trunk, accompanied by metabolic complications including insulin resistance, diabetes mellitus, and dyslipidemia [101,125]. The pathogenic mechanisms in FPLD2 strongly support the gene expression hypothesis. Mutant lamin A/C displays altered interactions with chromatin and transcriptional regulators. A central mechanism involves disrupted binding and sequestration of Sterol regulatory element-binding protein 1 (SREBP1), a key regulator of adipogenesis and lipid metabolism, at the nuclear periphery. This disruption impairs expression of genes required for adipocyte differentiation and lipid storage, leading to lipodystrophy. Structural analyses further indicate that residues frequently mutated in lipodystrophy (e.g., K486 and H506) are critical for lamin A/C interaction with nucleosomal DNA, linking structural perturbation to transcriptional dysregulation [20]. Notably, *LMNB2* has also been identified as a susceptibility gene for Acquired partial lipodystrophy (APL), expanding the genetic landscape of adipose tissue laminopathies [118]. Lamin B2 labeling also appears in the anterior horn and spinal cord neurons of postmortem Amyotrophic lateral sclerosis patients with frontotemporal dementia and G38R or D40G protein variants [126].

Peripheral neuropathies and leukodystrophies involve dysfunction of both A- and B-type lamins. Autosomal dominant axonal Charcot–Marie–Tooth disease type 2B1 is caused by *LMNA* mutations [14]. More prominently, B-type lamin dysfunction gives rise to severe neurological disorders. Duplication of the *LMNB1* gene, leading to its overexpression, causes Adult-onset autosomal dominant leukodystrophy (ADLD), a progressive degeneration of central nervous system white matter [108]. Studies indicate that lamin B1 overexpression exerts a toxic effect primarily on astrocytes rather than oligodendrocytes, reducing secretion of survival factors such as Leukemia inhibitory factor (LIF) and promoting inflammatory responses that contribute to demyelination [109]. Lamin B1 is an important regulator of brain development and aging. In the hippocampus of *Fmr1^Δ^^exon 8^* rats, decreased lamin B1 expression leads to nuclear abnormalities [116]. In contrast, biallelic loss-of-function mutations in the LBR cause a spectrum of disorders ranging from Pelger–Huët anomaly, characterized by abnormal neutrophil nuclear morphology, to severe skeletal dysplasias including Greenberg dysplasia. These findings highlight that LBR functions dually in cholesterol biosynthesis and heterochromatin tethering during skeletal development [127].

Progeroid syndromes represent the most severe laminopathies and are characterized by accelerated manifestation of multiple features associated with physiological aging. The prototypical disorder is HGPS, a sporadic dominantly acting disease caused by a de novo point mutation in *LMNA* (c.1824C>T, p.G608G) that activates a cryptic splice site. This mutation results in the production of progerin, a permanently farnesylated, truncated form of lamin A that lacks 50 amino acids [22,78]. Progerin accumulates within the nuclear lamina and causes pronounced nuclear morphological abnormalities, including nuclear envelope budding and lobulation, thickening of the nuclear lamina, and loss of peripheral heterochromatin [79]. Its accumulation initiates a cascade of cellular defects, including impaired DNA repair, genomic instability, progressive telomere dysfunction, disrupted mitotic progression, and altered stem cell maintenance [80,128]. Deficiency of *ZMPSTE24*, which prevents the final proteolytic processing of prelamin A, similarly results in accumulation of farnesylated prelamin A and causes restrictive dermopathy as well as milder progeroid syndromes, further supporting the toxicity of improperly processed lamin A precursors [84,129].

The laminopathy spectrum also includes overlapping and atypical phenotypes. MAD arises from homozygous *LMNA* mutations (e.g., R527H) and combines skeletal abnormalities with lipodystrophy and progeroid features [62]. Furthermore, some patients diagnosed with atypical Werner syndrome, which is classically associated with WRN mutations, harbor heterozygous *LMNA* variants, indicating substantial phenotypic convergence between distinct progeroid mechanisms [88].

**Figure 3:**
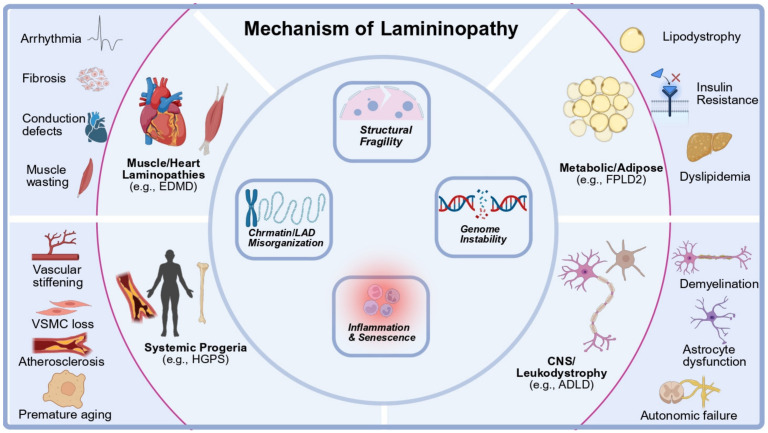
Nuclear Pathology and Organ-Level Clinical Spectra in Laminopathy Mechanisms. At the cellular level, lamin defects trigger interconnected pathomechanisms, including structural fragility, chromatin and lamin-associated domain (LAD) misorganization, genomic instability, and activation of inflammatory and senescence pathways. These nuclear abnormalities manifest as distinct clinical spectra depending on the affected tissue: muscle/heart laminopathies with conduction defects, arrhythmia, fibrosis, and muscle wasting; systemic progeroid syndromes featuring vascular stiffness, vascular smooth muscle cell loss, atherosclerosis, and premature aging; metabolic/adipose laminopathies manifesting as lipodystrophy, insulin resistance, and dyslipidemia; and central nervous system disorders associated with demyelination, astrocyte dysfunction, and autonomic failure.

Collectively, the laminopathy spectrum underscores the diverse consequences of nuclear envelope dysfunction. Although historically framed as competing structural and gene expression models, accumulating evidence indicates that these mechanisms are mechanistically intertwined and frequently act in concert. The specific tissue phenotype appears to emerge from the interaction between a given mutation and the mechanical, metabolic, and transcriptional environment of the affected tissue, as well as developmental timing [130]. A comprehensive understanding of this genotype–phenotype landscape remains essential for the development of targeted therapeutic strategies for laminopathies (Figure 3).

## 4. Laminopathies Linked to B-Type Lamins

### 4.1. Lamin B1 Overexpression in ADLD

ADLD represents a paradigm of B-type lamin dysfunction and is mechanistically distinct from most *LMNA*-associated diseases. ADLD is a fatal adult-onset neurodegenerative disorder characterized by progressive demyelination of the central nervous system, leading to autonomic dysfunction, motor impairment, and premature death. The genetic cause is a non-coding duplication of the *LMNB1* gene, resulting in approximately 1.5- to 2-fold overexpression of lamin B1 [108]. This observation establishes lamin B1 as a dosage-sensitive protein. Both its upregulation, as observed in ADLD, and its reduction during cellular senescence are associated with pathological states, suggesting that proper nuclear lamina function requires tightly regulated expression levels [110,113]. The primary cellular consequence of *LMNB1* overexpression involves nuclear toxicity. Patient-derived cells and experimental models exhibit abnormal nuclear morphology, including envelope invaginations and irregular nuclear contours, which are thought to contribute to broader cellular dysfunction [108]. However, a central unresolved question in ADLD is tissue specificity, given that ubiquitous *LMNB1* overexpression selectively affects central nervous system white matter. Recent studies shift attention from oligodendrocytes to astrocytes as primary contributors to disease pathogenesis. Evidence suggests that astrocytes are particularly sensitive to elevated lamin B1 levels. In vitro models indicate that *LMNB1* overexpression reduces secretion of LIF, a cytokine required for oligodendrocyte progenitor survival and differentiation. This finding supports a non–cell-autonomous mechanism in which astrocytic dysfunction compromises trophic support, thereby contributing to oligodendrocyte loss and demyelination. Furthermore, lamin B1 overexpression is associated with oxidative stress and activation of pro-inflammatory signaling pathways, including nuclear factor kappa B (NF-κB), which may further exacerbate the local neurotoxic environment [109]. Alterations in heterochromatin organization have also been reported and may contribute to dysregulation of genes involved in glial homeostasis [110].

Despite these advances, important mechanistic gaps remain. The molecular pathway linking *LMNB1* duplication to reduced LIF expression remains incompletely defined [109]. In addition, the delayed adult onset of ADLD, despite lifelong lamin B1 overexpression, suggests that age-dependent compensatory mechanisms or cumulative cellular damage thresholds may contribute to disease manifestation [113]. Consequently, therapeutic strategies aimed at modulating *LMNB1* expression, restoring trophic signaling, or reducing oxidative stress remain largely at the preclinical stage [109]. PubChem CID 662896 and CID 5308648 reduced *LMNB1* in a dose-dependent manner without causing cytotoxicity and corrected nuclear abnormalities associated with *LMNB1* overexpression. CID 662896 also reduced the level of *LMNB1* in fibroblast samples from ADLD patients, exhibiting good drug-like physicochemical properties and crossing the blood–brain barrier in mouse studies. It is expected to be a promising candidate for ADLD treatment [111].

### 4.2. Lamin B1 Dysfunction in Senescence and Aging

In contrast to the pathogenic overexpression observed in ADLD, reduction in lamin B1 represents an established hallmark of cellular senescence and physiological aging [110,113]. Accumulating evidence indicates that this decline functions not only as a biomarker but may also contribute functionally to aging-associated phenotypes. Reduced *LMNB1* expression disrupts nuclear lamina organization, resulting in increased nuclear fragility, altered nuclear morphology, and diminished resistance to mechanical stress [113]. One major consequence of lamin B1 loss is destabilization of peripheral heterochromatin. Lamins contribute to tethering transcriptionally repressive chromatin domains to the nuclear periphery. Loss of lamin B1 correlates with decreased heterochromatin-associated histone modifications, including trimethylation of histone H3 lysine 9 (H3K9me3), a common feature of senescent cells that is associated with transcriptional derepression and genomic instability [57,114]. These chromatin alterations are linked to activation of the Senescence-associated secretory phenotype (SASP), as the altered chromatin landscape permits expression of pro-inflammatory cytokines and related factors [115]. Evidence further suggests the existence of a reinforcing feedback loop in which chromatin disruption and DNA damage promote senescence-associated pathways that sustain lamin B1 downregulation [112].

The relationship between lamin B1 and aging is further complicated by its interplay with progerin, the mutant lamin A responsible for HGPS. Although progerin accumulation drives HGPS, low-level activation of the same cryptic splice site has been detected in normally aged tissues [44,46]. Age-associated progerin production may act synergistically with lamin B1 decline. Furthermore, telomere dysfunction, a key driver of replicative senescence, can induce progerin production, which may in turn exacerbate nuclear defects and contribute to further telomere damage and lamin disorganization [45,128,131]. This relationship suggests the existence of a self-reinforcing cycle linking nuclear lamina deterioration with fundamental aging processes.

However, lamin B1 function during aging is not uniform across cell types. For example, its expression pattern during neurogenesis is conserved in mammals but differs in certain non-mammalian vertebrates, suggesting evolutionary divergence in its regulatory roles [132]. Moreover, the upstream signals responsible for lamin B1 reduction during senescence—whether persistent DNA damage, oxidative stress, or transcriptional reprogramming—remain incompletely understood. Clarifying these mechanisms is essential for distinguishing whether lamin B1 loss primarily drives aging phenotypes or represents a downstream consequence of other cellular stresses.

### 4.3. Lamin B2 Dysregulation in Metabolism and Cancer

Compared with *LMNB1*, disease associations involving *LMNB2* remain less clearly defined but suggest distinct and context-dependent functions. Lamin B2 contributes to nuclear architecture as part of a B-type lamin network and appears to participate in the spatial organization of constitutive heterochromatin, distinguishing its role from lamin B1, which is more frequently associated with facultative heterochromatin [26,53]. Its pathological relevance has been most frequently reported in metabolic disease and cancer, although mechanistic understanding remains limited.

In adipose tissue disorders, *LMNB2* has been identified as a susceptibility gene for APL. Genetic analyses identified heterozygous variants in *LMNB2* (e.g., p.R215Q and p.A407T) in affected individuals, suggesting that lamin B2 dysfunction may predispose to adipose tissue loss [118]. The underlying molecular mechanisms remain uncertain. It has been proposed that altered nuclear mechanics or disrupted chromatin interactions in preadipocytes may impair differentiation or survival, but functional validation of these variants remains limited [117,118].

The role of lamin B2 in cancer appears complex and context-dependent. *LMNB2* expression is dysregulated across multiple malignancies, with divergent functional consequences depending on tumor type [117]. Increased expression has been reported in cancers such as colorectal and prostate cancer, where it may enhance nuclear mechanical stability or influence proliferation-associated transcriptional programs [24,110]. In contrast, other studies indicate that lamin B2 supports accurate mitotic spindle assembly and chromosome segregation [24]. Loss or dysfunction of lamin B2 can therefore promote mitotic errors and genomic instability. These alterations may contribute to tumorigenesis in some contexts but may also limit tumor growth if genomic instability exceeds tolerable thresholds. This duality underscores the importance of cellular context in determining whether lamin B2 functions as a facilitator of tumor progression or as a stabilizer of genomic integrity. Furthermore, its essential role in DNA replication adds another layer of complexity to its function in rapidly dividing cancer cells [69,117].

Several key questions remain unresolved. The mechanisms by which specific *LMNB2* mutations alter chromatin tethering or mitotic processes are not fully defined. The basis for tissue-specific disease associations, particularly in adipose tissue, also remains unclear. Finally, the extent of functional redundancy between lamin B1 and lamin B2, especially under pathological conditions, remains poorly understood and complicates attribution of disease phenotypes to individual isoforms [3,110].

## 5. Laminopathies Linked to A-Type Lamins

### 5.1. Muscle Laminopathies from Nuclear Instability

Striated muscle—comprising cardiac and skeletal muscle—is exquisitely sensitive to *LMNA* mutations, giving rise to a spectrum of disorders including EDMD, Limb-girdle muscular dystrophy type 1B (LGMD1B), and *LMNA* mutation-related DCM. These tissues endure immense and constant mechanical stress, placing unparalleled demands on nuclear integrity and mechanical signaling. Accordingly, the pathogenic mechanisms, while sharing common themes of nuclear fragility and aberrant signaling, manifest with distinct clinical severities and patterns, highlighting the complex interplay between mutation type and cellular context [15,23,133].

EDMD is the prototypical disorder supporting the structural hypothesis of laminopathies. It presents with the classic triad of early joint contractures, progressive muscle weakness in a humero-peroneal distribution, and cardiac conduction defects [91,92]. Caused by mutations in *LMNA* or *EMD* (encoding emerin), the pathophysiology converges on a defective LINC, compromising mechanical coupling between the cytoskeleton and nucleus. A weakened nuclear lamina succumbs to the forces of muscle contraction, leading to recurrent nuclear envelope rupture, herniation of chromatin, and DNA damage, which ultimately trigger p53-mediated apoptosis or senescence in muscle fibers [34]. Recent studies further identify Net39, a muscle-specific nuclear envelope protein, as a critical protector. The loss of Net39 phenocopies EDMD, while AAV (adeno-associated virus)-mediated gene therapy rescues nuclear morphology and function in *Lmna*-deficient mice, positioning it as a key component in safeguarding nuclei from mechanical stress [35].

LGMD1B typically manifests with later-onset, proximal limb-girdle weakness and variable cardiac involvement. The distinction from EDMD is often blurred, as identical *LMNA* mutations can cause either phenotype within a single family, underscoring the influence of genetic modifiers or environmental factors on disease expression [77]. The pathogenic mechanisms are presumed to overlap significantly with EDMD, involving nuclear instability and defective mechanical signaling. However, the precise determinants that steer pathology toward a proximal limb-girdle pattern of weakness, in contrast to the humero-peroneal distribution observed in EDMD, remain largely unknown. *LMNA* mutations can also cause Congenital muscular dystrophies (CMD) to be a phenotype- and genotype-heterogeneous disease. The p.R249W mutation in *LMNA*-related CMD is associated with early loss of walking ability [93].

*LMNA* mutation-related DCM represents the most severe and prevalent striated muscle laminopathy, characterized by ventricular dilation, systolic dysfunction, and a disproportionately high risk of malignant arrhythmias and sudden cardiac death. Prognosis is worse than for other genetic forms of DCM, indicating a uniquely aggressive disease course [106,107,121,122,123,134]. In this context, structural failure converges with profound signaling dysregulation. A dominant theme is the constitutive activation of stress-responsive pathways, particularly the MAPK/ERK cascade, which drives pathological gene expression, fibrosis, and apoptosis in cardiomyocytes [90]. This signaling derangement is mechanistically linked to disrupted nucleo-cytoskeletal coupling. Mutant lamins promote ERK-dependent phosphorylation of cofilin-1, which sequesters the transcription factor MRTF-A in the cytoplasm. This impairs SRF-mediated expression of genes such as ATAT1, leading to decreased α-tubulin acetylation, mislocalization of connexin-43, and arrhythmogenesis [63]. E161K and K97E mutation of lamin A/C have been widely reported in patients with DCM. These mutations can lead to large-scale destruction of the peripheral layer and the subsequent formation of heterochromatin tissue, as well as the formation of aggregates within the nucleoplasm [89].

Beyond MAPK activation, mutant lamins induce pervasive cellular stress. In cardiomyocytes, lamin deficiency can cause nuclear envelope rupture and subsequent Golgi apparatus fragmentation, triggering a specialized Golgi stress response and the integrated stress response via activating transcription factor 4 (ATF4)–CCAAT/enhancer-binding protein homologous protein (CHOP) signaling, thereby promoting cell death [34]. Furthermore, mitochondrial dysfunction emerges as a key intermediary. Lamin A/C haploinsufficiency accelerates degradation of the deacetylase SIRT1, leading to mitochondrial reactive oxygen species accumulation. This activates calcium/calmodulin-dependent protein kinase II (CaMKII), which phosphorylates the ryanodine receptor2 (RYR2)—a trigger for arrhythmias—and stabilizes SUN1 protein, thereby exacerbating nuclear envelope defects [65].

The cardiac selectivity is further illuminated by mutation-specific mechanisms that disrupt cardiac transcriptional programs. For instance, the *LMNA* Q353R mutation causes DCM by aberrantly trapping TEAD1 at the nuclear periphery. TEAD1 is a key effector of the Hippo pathway, which mediates mechanical signaling, and its sequestration disrupts normal cardiac gene expression. This sequestration prevents TEAD1 from activating genes essential for cardiac maturation and function. Notably, the same Q353R mutation was shown in a separate study to impair DNA repair by sequestering the VDR, blunting the expression of DNA repair genes; vitamin D supplementation rescued this defect in cellular models [64]. These findings underscore how specific mutations can disrupt tissue-critical transcriptional networks.

Persistent gaps in understanding striated muscle laminopathies remain significant. The fundamental genotype–phenotype conundrum persists: why do different *LMNA* mutations produce markedly distinct clinical manifestations despite shared structural defects? The role of genetic background and epigenetic modifiers is suspected but remains poorly defined. Furthermore, the initial molecular event linking mutant lamin proteins at the nuclear envelope to cytoplasmic kinase activation, such as ERK signaling, remains incompletely understood [90]. Therapeutic translation therefore remains challenging. While preclinical models show promise for interventions such as p38 MAPK inhibitors or histone deacetylase 6 (HDAC6) inhibitors, developing effective and safe treatments for patients—particularly for aggressive *LMNA* mutation-related DCM—remains an ongoing challenge [135].

### 5.2. Adipose Tissue Laminopathies and Metabolic Dysregulation

In stark contrast to the mechanically driven pathologies of muscle, laminopathies affecting adipose tissue—most notably FPLD2—provide strong support for the gene expression hypothesis [103]. FPLD2 is an autosomal dominant disorder characterized by progressive, post-pubertal loss of subcutaneous adipose tissue from the extremities, gluteal region, and trunk, with paradoxical fat accumulation in the face and neck. This aberrant fat distribution is accompanied by severe metabolic sequelae, including insulin resistance, diabetes mellitus, hepatic steatosis, and dyslipidemia, conferring a high risk of cardiovascular disease [101,125,136]. FPLD2 is almost exclusively caused by heterozygous missense mutations in *LMNA*, with a striking cluster of mutations (e.g., R482W/Q/L, K486N) located within the immunoglobulin-like fold of the lamin A/C tail, a domain critical for protein–protein and protein–DNA interactions [100,104].

The pathogenesis centers on disruption of adipogenic transcriptional programs. The mutant lamin A/C protein retains its ability to incorporate into the nuclear lamina but exhibits altered binding properties. A key mechanism involves sequestration of SREBP1 at the nuclear periphery [105]. SREBP1 is a master transcription factor regulating genes essential for adipocyte differentiation, lipid synthesis, and glucose metabolism. By trapping SREBP1, mutant lamins prevent its proper genomic localization and transactivation function, thereby impairing adipogenesis and promoting lipoatrophy. This mechanistic framework is supported by structural evidence. Cryogenic electron microscopy studies reveal that residues frequently mutated in FPLD2, such as K486 and H506, directly participate in lamin A/C interaction with nucleosome DNA [20,48]. Furthermore, lamin A/C, in complex with BAF, can bridge two nucleosomes and influence higher-order chromatin structure [20]. Mutations such as R482W may disrupt these chromatin interactions, leading to dysregulation of adipose-specific gene networks without inducing the severe nuclear fragility observed in muscular dystrophies. Lamin A/C is a key regulator of cysteine catabolism flux. Regulating cystathionine γ- lyase and cystathionine β- synthase can alleviate the aging phenotype caused by lamin A/C mutations, rescue abnormal cell fate and function, restore DNA damage repair ability, and highlight the potential of regulating cell metabolism to alleviate epigenetic diseases [137,138].

The metabolic complications of FPLD2 extend beyond adipose tissue and involve systemic metabolic reprogramming. Patients often exhibit muscle hypertrophy yet display reduced muscle strength and increased fatigability. Studies report impaired mitochondrial oxidative phosphorylation, particularly reduced fatty acid utilization, accompanied by transcriptional signatures indicative of reduced mitochondrial biogenesis and protein catabolism pathways, suggesting features resembling accelerated tissue aging [125]. These findings indicate that metabolic dysfunction in FPLD2 is not confined to adipocytes but exerts secondary effects on other insulin-sensitive tissues.

The tissue specificity of FPLD2 remains incompletely understood. It has been proposed that adipocytes, which undergo extensive nuclear remodeling during differentiation, may be uniquely dependent on precise lamin–chromatin interactions for transcriptional regulation. The specific requirement for SREBP1 activity in adipogenesis may therefore render this cell type particularly sensitive to its functional sequestration by mutant lamins [9,105]. Notably, the role of B-type lamins in adipose biology is also emerging, with *LMNB2* identified as a susceptibility gene for APL, suggesting overlapping yet distinct pathogenic mechanisms within the nuclear lamina.

Significant gaps persist in the FPLD2 field. While sequestration of SREBP1 represents a central mechanism, the full repertoire of transcription factors and chromatin regulators affected by FPLD2-causing lamin mutations remains incompletely defined [9]. Furthermore, the post-pubertal onset of disease suggests hormonal modulation; however, the interplay between sex hormones, lamin function, and adipose biology remains poorly understood. Most critically, effective targeted therapies remain lacking. Current management focuses primarily on controlling metabolic complications rather than correcting the underlying nuclear defect, highlighting the need for mechanism-based therapeutic strategies [104,105].

Mutations in nuclear-layer-related genes, including *LMNA*, are associated with metabolic-associated fatty liver disease (MASLD), and changes in the nuclear membrane of MASLD patients are caused by downregulation of *ZMPSTE24*. In addition, *Zmpste24* mutant mice exhibit hepatic steatosis and upregulation of p53 target genes. Functional analysis determined that p53 is a regulator of FOXA2 differential expression and binding genes in MASLD patients. In male MASLD patients, mir-141-3p inhibited the expression of *ZMPSTE24*.

### 5.3. Progeroid Laminopathies and Mechanisms of Premature Aging

Progeroid laminopathies, in which defects in nuclear lamina components lead to accelerated aging phenotypes, provide an important framework for examining the role of nuclear architecture in organismal aging. The most severe and extensively studied condition in this category is HGPS, a condition that has become synonymous with the study of premature aging. Its molecular cause, progerin—a mutant form of lamin A—has been extensively characterized and is associated with cellular defects that extend from the nuclear envelope to systemic pathology. Furthermore, MAD and *LMNA*-related atypical Werner syndrome represent variations on this theme, offering comparative insights into how different lamin perturbations lead to overlapping yet distinct progeroid outcomes [120,139].

Although there are other mutation sites that can cause HGPS [82], it is more commonly caused by a single, spontaneous point mutation in the *LMNA* gene (c.1824C>T, p.G608G) [78,81]. This silent mutation generates a cryptic splice donor site within exon 11, resulting in an mRNA lacking 150 nucleotides. The translated protein, progerin, lacks 50 amino acids near its C-terminus, including the cleavage site for the zinc metalloprotease *ZMPSTE24* [22]. Under physiological conditions, *ZMPSTE24* catalyzes the final proteolytic step in lamin A maturation by removing the farnesylated C-terminal tail of prelamin A, thereby generating mature lamin A. In contrast, progerin retains this farnesyl modification, which promotes persistent association with the INM and disrupts normal lamin polymer organization [80,83].

**Figure 4:**
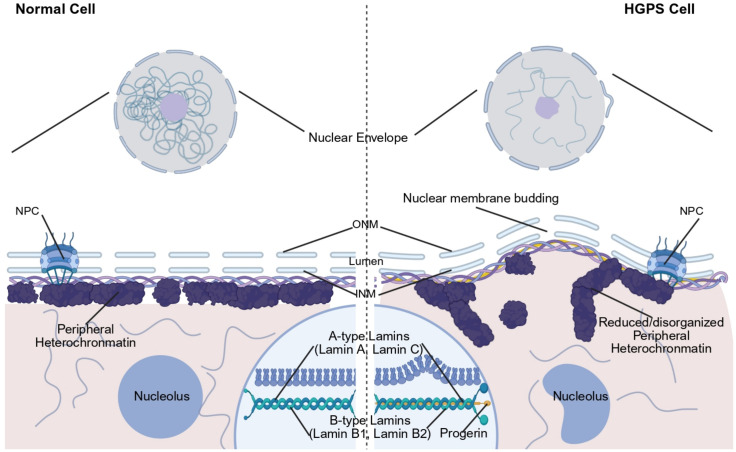
Comparison of nuclear envelope and nuclear lamina architecture in normal and Hutchinson-Gilford progeria syndrome (HGPS) cells. In normal cells, nuclear envelopes are supported by an organized nuclear lamina, comprising lamin A/C and lamin B1/B2. Peripheral heterochromatin is properly enriched at the nuclear rim. In HGPS cells, accumulation of progerin disrupts A-type lamin organization. This leads to nuclear membrane remodeling, loss or disorganization of peripheral heterochromatin, and broader structural defects, including altered nucleolar organization.

The cellular effects of progerin are consistent with a dominant-negative mechanism affecting multiple nuclear processes. One of the earliest observable phenotypes is altered nuclear morphology and mechanics. Progerin accumulation leads to misshapen nuclei characterized by blebs, lobulations, and thickening of the nuclear lamina, which correlate with increased nuclear stiffness rather than purely morphological abnormalities [79]. Notably, reduced nuclear deformability is associated with impaired cell migration in confined environments, suggesting that altered nuclear mechanics may contribute to defects in tissue repair and homeostasis [8] (Figure 4). The occurrence of nuclear membrane wrinkling induced by progerin, which regulates cytoskeletal tension, is controlled by the molecular interaction between SUN1 and lamins, which controls myosin-dependent nuclear tension attenuation. Nuclear deformation specifically regulates gene expression related to premature aging by modifying mechanosensitive signaling pathways. The expression of progerin disrupts the morphology of the endogenous lamin B1 network structure, causing irregularities and large openings. Increased lamin B1 expression reverses the morphological abnormalities of progerin network structure and significantly reduces the frequency of nuclear envelope rupture and bubbles. Therefore, the expression of progerin disrupts the overall structure of the nuclear layer, but this effect, as well as nuclear envelope rupture and bubbles, can be eliminated by increasing the expression of lamin B1 [140].

At the molecular level, progerin promotes genomic instability through multiple converging mechanisms. Studies demonstrate that DNA damage repair pathway choice shifts toward error-prone NHEJ at the expense of homologous recombination, thereby increasing mutational burden [11]. Consequently, HGPS cells exhibit widespread DNA hypomethylation, loss of heterochromatin-associated marks such as H3K9me3, and detachment of LADs from the nuclear periphery [61,141,142]. These epigenetic alterations are associated with transcriptional dysregulation and activation of senescence-associated gene programs.

Evidence indicates that nuclear envelope budding causes genomic material loss via autophagic degradation of extruded chromatin and telomeres [143,144]. A recent study identified a novel pathogenic mechanism in HGPS, which, working in concert with lamin A/C, induces abnormal nuclear envelope budding. This process transports chromatin and telomeres into the cytoplasm, where they are subsequently cleared by autophagy, leading to progressive telomere loss and accelerated aging; emerin antagonizes this process by maintaining nuclear envelope integrity [144]. Importantly, the natural compound chaetocin sequesters progerin from the nuclear envelope by sustaining ERK1/2 activation (partially via downregulating DUSP6), thereby inhibiting nuclear envelope budding, preventing chromatin and telomere loss, alleviating progeria-associated defects, and extending the lifespan of HGPS mice. These findings highlight nuclear envelope budding as a critical driver of premature aging and identify chaetocin as a promising therapeutic candidate for HGPS.

Progerin additionally perturbs cellular signaling and metabolic regulation. Studies report disrupted nucleocytoplasmic transport, including cytoplasmic retention of the histone acetyltransferase p300, which interferes with feedback regulation of mechanistic target of rapamycin complex 1 (mTORC1) signaling and impairs autophagy [145]. Treatment with mTOR inhibitors can alleviate the damage induced by progerin [146]. Furthermore, progerin persistence on membranes during mitosis delays nuclear envelope reassembly and cytokinesis, resulting in binucleation and aneuploidy in some cells [80]. These cumulative stresses promote cellular senescence and depletion of progenitor populations. Importantly, emerging evidence indicates that progerin toxicity may not be strictly cell-autonomous; expression in a limited fraction of tissue-resident cells can induce tissue-wide degeneration through paracrine senescence-associated signaling [147]. Additionally, progerin impairs cellular autophagy. In HGPS cells, nuclear-localized progerin is released into the cytoplasm via nuclear envelope budding and degraded through autophagy. Lysosomal defects in HGPS cells compromise progerin clearance. HGPS cells exhibit pronounced lysosomal defects, which impair the autophagic clearance of progerin expelled from the nucleus via nuclear envelope budding. Activating lysosome biogenesis through PKC stimulation or mTORC1 inhibition restores progerin degradation, alleviates DNA damage and SASP, revealing that progerin disrupts lysosomal metabolic regulation and that enhancing lysosome function can counteract premature aging [148].

At the organismal level, these cellular defects manifest as progressive multisystem degeneration, including alopecia, subcutaneous fat loss, skeletal abnormalities, and severe cardiovascular disease. Patients typically die during adolescence due to myocardial infarction or stroke. Vascular pathology is characterized by accelerated atherosclerosis and prominent adventitial fibrosis [97]. The mechanism involves loss of vascular smooth muscle cells and endothelial dysfunction. Recent studies suggest that altered mechanical signaling contributes to endothelial dysfunction, potentially through YAP/TAZ pathway activation secondary to increased cellular and extracellular stiffness [149]. Importantly, low-level progerin production has been detected in normal aging tissues, raising the possibility that similar mechanisms may contribute to physiological vascular aging, although this relationship remains incompletely defined [44,150].

Therapeutic strategies targeting progerin have evolved substantially. The farnesyltransferase inhibitor lonafarnib reduces progerin membrane association, improves nuclear morphology, and extends median survival by approximately 2.5 years, although alternative prenylation limits therapeutic efficacy [21]. More recently, adenine base editing (ABE) approaches correcting the causative *LMNA* mutation in mouse models restored vascular integrity and significantly extended lifespan, highlighting the potential of genome editing strategies [98]. Additional approaches aim to disrupt pathogenic protein interactions or inhibit downstream processes such as nuclear envelope budding [35].

Restrictive skin disease (RD) is a rare and fatal congenital autosomal recessive inherited syndrome of vertebral plate disease, characterized by systemic tight translucent skin, deformities, multiple congenital joint abnormalities, and pulmonary hypoplasia. It is caused by mutations in *ZMPSTE24* or *LMNA*. The mutations in these genes can disrupt the production of lamin A, thereby disrupting the structural integrity of the nuclear membrane, and may also lead to survivable syndromes such as mandibular dysplasia. The longest survival period of RD is 120 days [85]. In mandibular dysplasia, a homozygous pathogenic frameshift variant in *ZMPSTE24*, c.1085dup (p.L362Ffs*19), has been identified, indicating that similar mutations in the *LMNA* gene lead to different phenotypes [85].

MAD, caused by distinct *LMNA* mutations (e.g., homozygous R527H), presents a milder progeroid phenotype involving skeletal dysplasia and partial lipodystrophy while generally sparing severe early cardiovascular decline [62,86,151]. Evidence suggests that disease severity correlates with hierarchical chromatin disorganization, including altered LAD positioning and shortening of TADs, leading to dysregulation of tissue maintenance genes [61]. The substitution of the L648R single amino acid can block the maturation of prelamin A in mice, thereby simulating skull deformities caused by abnormal suture fusion, similar to those in patients with craniosynostosis. The accumulation of prelamin A is associated with multiple suture synapses with low bone density, and this mutation disrupts the stemness of bone stem cells and subsequent stem cell-mediated osteogenic proliferation and differentiation. Comparing gene expression profiles further revealed the cytoskeletal dynamics associated with aging and smooth suturing of bone-generating cells in mice and humans. Functional studies have shown that the abnormal structure of precursor cell nuclei caused by the accumulation of proline A can affect the necessary cytoskeleton tissue and nucleoskeleton assembly for craniofacial bone development [87].

A profound comparative insight comes from analyzing HGPS alongside Werner syndrome, a classic progeria caused by loss-of-function mutations in the WRN DNA helicase. This comparison highlights the principle of convergent pathological pathways. Although the primary defects are distinct—structural (such as progerin) versus metabolic (such as DNA repair orhelicase)—both syndromes converge on downstream hallmarks like heterochromatin loss, stem cell exhaustion, and chronic inflammation [152,153]. However, they exhibit divergent kinetics of aging. Isogenic stem cell models reveal that Werner syndrome mesenchymal stem cells undergo an early-onset, gradual decline, whereas HGPS counterparts show a late-onset but precipitous functional collapse, indicating different paces toward a shared senescent endpoint [154]. Clinically, this convergence is evident in atypical Werner syndrome, where a subset of patients with a Werner syndrome -like phenotype are found to harbor heterozygous *LMNA* mutations instead of WRN mutations, blurring diagnostic boundaries [88]. A critical unresolved paradox is that Werner syndrome patients have a high incidence of cancers, while HGPS patients do not, despite both having genomic instability. This suggests progerin may activate potent tumor-suppressive checkpoints or that the nature of the DNA damage it induces is less oncogenic [155]. Major challenges remain in understanding the determinants of phenotypic variability, defining progerin’s precise role in normal aging, and safely translating groundbreaking therapies like in vivo base editing into clinical practice.

## 6. Experimental Models and Treatment Strategies

### 6.1. Laminopathies Animal Models

Animal models, particularly genetically engineered mice, are indispensable for unraveling the in vivo pathophysiology of laminopathies and serve as critical platforms for preclinical therapy development [156,157]. These models are primarily generated to recapitulate specific human genetic lesions. For progeroid syndromes, the *Lmna*^G609G/G609G^ mouse, which carries the orthologous HGPS mutation, is a well-recognized model. It manifests key disease features including growth retardation, bone abnormalities, alopecia, loss of vascular smooth muscle cells, and significantly shortened lifespan, and has been extensively used to evaluate therapies ranging from farnesyltransferase inhibitors to advanced genetic corrections like base editing [98,99,158]. Earlier models such as the *Lmna*^L530P/L530P^ and *Zmpste24*^-/-^ mice, which accumulate mutant or unprocessed prelamin A, respectively, established the foundational link between defective lamin A processing and accelerated aging phenotypes [159]. For cardiac and muscular laminopathies, models like the *Lmna*^H222P/H222P^ mice develop DCM and conduction defects, faithfully mirroring human *LMNA* mutation-related DCM and elucidating pathogenic signaling cascades, including MAPK/ERK hyperactivation [63,90]. In the mouse model of Ruijs Alfs syndrome associated with SPRTN mutations, a large amount of unrepaired DNA protein crosslinks and micronuclei were accumulated in the body, exhibiting strong innate immune activation. Many mice die in the early stages, while surviving individuals exhibit a series of premature aging characteristics, including small body size, craniofacial deformities, fat metabolism disorders, premature hair whitening, and some abnormalities are already apparent during embryonic development. By genetic or pharmacological means, inhibiting the cGAS-STING pathway from early developmental stages significantly reduces embryonic lethality in mice and improves premature aging-related symptoms [160]. Despite their utility, murine models present significant limitations that challenge direct translation to human disease. A major issue is the differences in lifespan and disease phenotypes: progeroid mice often die within weeks from complications such as muscle wasting, whereas human HGPS patients typically die in their teens primarily from atherosclerosis, making it difficult to model long-term vascular progression [159,161]. Species-specific differences in telomere biology, metabolism, and immune function further modulate disease manifestation and potential therapeutic responses [157,162]. Additionally, many studies employ young, inbred, genetically identical mice, which fail to capture the aged tissue microenvironment and genetic heterogeneity present in human patients, potentially overestimating treatment efficacy [162]. Furthermore, the field increasingly advocates for genetically heterogeneous mouse stocks or naturally aged cohorts, although these approaches are more resource-intensive [157]. To better approximate human populations, Liu et al. used the BE4max adenine base editor to generate the first primate progeria model harboring a single-base mutation. HGPS monkeys expressed the toxic form of lamin A, progerin, and exhibited phenotypes including growth retardation, bone alterations, and vascular abnormalities. Thus, this primate model genetically and clinically mimics HGPS in humans, which will strongly facilitate research on pathogenesis and therapeutic strategies [163].

Other model organisms provide complementary insights. *Drosophila melanogaster* and *Caenorhabditis elegans* offer unparalleled genetic tractability and short lifespans for rapid, large-scale screens to identify genetic modifiers and evolutionarily conserved pathways relevant to nuclear envelope function and aging [36,152]. For Werner syndrome, Wrn-deficient mice have been developed, though they frequently require a concurrent telomerase deficiency to fully recapitulate the human progeroid spectrum, underscoring the complex interplay between nuclear lamina integrity, DNA repair, and telomere homeostasis [152,153]. Zebrafish is also one of the important models for studying laminopathies [164]. Using the *lmna* zebrafish model for drug screening, L-carnitine treatment rescued impaired muscle endurance in *lmna* L35P zebrafish, whereas creatine administration restored muscle endurance in the *lmna* R453W mutant model. Mechanistically, creatine activates the AMPK and mTOR signaling pathways, thereby improving muscle endurance and swimming ability in *lmna* R453W zebrafish [95].

A key point is that animal models are fundamental for establishing causality, exploring tissue-specific mechanisms, and conducting initial therapeutic safety and efficacy studies. However, their predictive power for human clinical outcomes is inherently constrained by biological differences and often simplified disease contexts. Consequently, data derived from animal studies must be integrated with findings from human cell-based models to construct a robust and translatable framework for understanding and treating laminopathies [135,139].

### 6.2. iPSC-Based Models for Laminopathy Research

Patient-derived induced pluripotent stem cell (iPSC) technology has greatly advanced laminopathy modeling, offering a human genetic platform for mechanistic study and therapeutic screening [135,143,165,166,167,168]. By reprogramming somatic cells from patients with specific *LMNA* or *LMNB* mutations, researchers can generate an unlimited supply of disease-relevant cell types, including cardiomyocytes, adipocytes, vascular smooth muscle cells, and mesenchymal stem cells, which are often the primary affected cells in these disorders. For example, iPSC-derived cardiomyocytes from patients with *LMNA* mutation-related DCM recapitulate key pathological features such as nuclear envelope budding, arrhythmic calcium handling, apoptosis, and aberrant activation of MAPK/p38 signaling pathways, allowing for the direct testing of pathway-specific inhibitors [64,165]. Similarly, iPSC-derived models have been crucial for studying progeroid syndromes, revealing mutation-specific defects in chromatin organization, DNA repair, and stem cell differentiation kinetics [61,114].

A major strength of the iPSC platform is its utility for high-throughput drug screening and personalized therapeutic discovery. By generating isogenic control lines via gene correction, researchers can attribute phenotypic differences solely to the disease-causing mutation, creating a clean system for compound screening. This approach has identified potential therapeutic agents, such as Vitamin D for rescuing DNA repair deficits in cardiomyocytes carrying the *LMNA* Q353R mutation, and HDAC6 inhibitors for correcting microtubule-dependent defects in other *LMNA* cardiomyopathies [63,64]. Furthermore, iPSCs enable the modeling of developmental aspects of disease. Studies have shown that some *LMNA* cardiomyopathy mutations disrupt epigenetic programs during early cardiac lineage specification, suggesting a developmental origin for the disease that can be investigated and potentially modulated in differentiating iPSCs [130].

However, the iPSC platform is not without significant limitations. The most prominent is the immaturity of differentiated cells. iPSC-derived cardiomyocytes, for instance, exhibit a fetal-like gene expression profile, structural immaturity, and metabolic properties distinct from adult human cardiomyocytes, which may limit their ability to model late-onset, adult-stage disease phenotypes accurately [135]. Furthermore, the lack of a physiological tissue microenvironment—including mechanical forces, heterotypic cell–cell interactions, and systemic hormonal signals—in conventional 2D culture is a critical shortcoming. This is particularly relevant for laminopathies, where mechanical signaling is a central pathogenic mechanism [7,34]. To address this, advanced engineered platforms are being integrated. These include 3D engineered heart tissues or organoids that better mimic tissue-level mechanics and architecture, and microfluidic devices that subject cells to physiological shear stress or cyclic strain [8,149]. The combination of patient-specific iPSCs with these bioengineered systems represents the next frontier, creating more physiologically relevant human models to study how mutant lamins disrupt function in a context that approximates native tissue.

In conclusion, patient-derived iPSCs and associated engineered platforms provide an essential, human-centric complement to animal models. They offer unparalleled access to human genotypes for mechanistic dissection and drug discovery, particularly for rare mutations. Yet, the field must continue to innovate in driving cellular maturation and reconstructing tissue complexity to fully realize the translational potential of these models for developing effective therapies for laminopathies.

### 6.3. Therapeutic Strategies for Laminopathies

Therapeutic development for laminopathies has progressively shifted from symptomatic management toward interventions that directly mitigate the toxic effects of mutant lamins or correct their underlying molecular defects. Rather than following a single linear trajectory, current strategies span multiple levels of intervention, including modulation of lamin post-translational processing, attenuation of downstream cellular damage, disruption of pathogenic protein interactions, and direct genetic correction.

The first breakthrough emerged from understanding the critical role of protein farnesylation in the toxicity of progerin and prelamin A. This led to the repurposing and clinical testing of farnesyltransferase inhibitors, such as lonafarnib. In HGPS, lonafarnib reduces the membrane association of progerin, ameliorates nuclear morphology, improves vascular stiffness, and has been shown to extend median survival by approximately 2.5 years, culminating in its FDA approval—the first disease-modifying therapy for a progeroid syndrome [21]. However, farnesyltransferase inhibitors are not specific to progerin and affect prenylation of other proteins, potentially contributing to side effects. Furthermore, cells can employ alternative prenylation pathways to modify progerin, limiting the therapy’s comprehensiveness [90]. This has spurred the search for more specific approaches, such as dual prenylation inhibitors or molecules that block the cryptic splicing event that generates progerin mRNA.

Beyond targeting progerin’s maturation, significant effort is directed at inhibiting pathogenic downstream signaling cascades that are hyperactivated across multiple laminopathies. In this context, pathway-targeted therapies are evaluated primarily as pharmacological tools to restore cellular homeostasis. For example, inhibitors of stress-activated kinases or mTOR signaling are assessed for their ability to reduce apoptosis, fibrosis, or proteostatic imbalance in disease models, independent of their mechanistic origins [90,135,145]. Similarly, compounds targeting endothelial dysfunction or vascular stiffness are explored as means to ameliorate the dominant clinical manifestations of progeroid laminopathies, particularly cardiovascular disease.

More direct strategies target mutant proteins or deleterious interactions. Unique progerin C-terminal peptide blocks pathological sequestration of mitotic regulator BUBR1 by progerin, improving healthspan in HGPS mice [35]. Chaetocin inhibits progerin-induced nuclear envelope budding, reducing chromatin loss and extending lifespan in a progeria model [144]. Treating HGPS fibroblasts with selinexor can alleviate aging and promote the clearance of progerin through autophagy, while restoring the expression of many differentially expressed genes at the transcriptional level and rescuing aging-related cellular processes [169]. While highly innovative, these molecular interventions often face challenges related to drug delivery, stability, and potential off-target effects of the small molecules identified.

Gene therapy and genome editing offer potentially curative approaches by correcting genetic defects. For recessive disorders or haploinsufficiency, gene replacement via viral vectors is viable, as explored for Net39 in muscular dystrophy models [35]. Dominant disorders, including HGPS and lamin-related diseases, require allele-specific silencing or correction. RNA interference has shown efficacy in cells. However, the watershed moment came with the application of ABE. In a landmark study, a single systemic injection of ABE reagents packaged in AAV9 effectively corrected the pathogenic *Lmna* c.1827C>T (p.G609G) mutation in a mouse model of HGPS with ~20–60% efficiency in various tissues. This intervention dramatically rescued vascular pathology, reduced fibrosis, and extended median lifespan from 215 to 510 days, demonstrating the profound potential of in vivo genetic correction [98]. The ABE method for the *LMNA* gene variant R249Q and the cytosine base editing (CBE) strategy for the L35P variant were used to accurately correct and successfully rescue the pathological phenotype and prolong the lifespan of mice with *LMNA* L35P and R249Q mutations [94].

CRASP seq combines CRISPR-based genetic perturbation with deep sequencing of splicing reporter genes, revealing ZNF207 as a regulator of premature-aging protein splicing. ZNF207 depletion enhances typical *LMNA* splicing and reduces the level of progerin protein in patient-derived cells. The zinc finger domain of ZNF207 extensively affects alternative splicing by directly interacting with U1 small nuclear ribonucleoprotein (snRNP) components [170].

Despite its promise, the gene editing landscape is fraught with translational hurdles. For viral-delivered editors, long-term risks include vector genomic integration, immune responses to bacterial-derived editing proteins, and potential off-target genome editing. The HGPS base editing study itself noted a concern regarding liver tumor formation in long-lived treated mice, underscoring the need for rigorous safety profiling [98]. Furthermore, efficient and safe delivery to all relevant tissues, especially post-mitotic cardiomyocytes and skeletal muscle, remains a significant technical challenge. For editing approaches, the choice between NHEJ-mediated disruption of the mutant allele and homology-directed repair-mediated precise correction must be carefully weighed for each mutation and cellular context.

Taken together, the therapeutic landscape for laminopathies is rapidly diversifying and advancing. The progression from broad pathway inhibitors to precise genetic surgery mirrors the field’s growing molecular sophistication. The future likely lies in combination strategies—perhaps using a splicing modulator or farnesyltransferase inhibitor to reduce toxic protein load in the short term, alongside a one-time gene editing treatment for a permanent cure. Successfully navigating the remaining challenges of delivery, specificity, and long-term safety will be paramount to translating these remarkable preclinical advances into effective therapies for patients.

## 7. Conclusions and Outlook

Lamins and their associated nuclear envelope proteins are now recognized as dynamic organizers of nuclear function rather than static structural components. Across diverse experimental contexts, converging evidence supports a model in which the nuclear lamina integrates mechanical resilience, chromatin organization, genome maintenance, and stress-response wiring. Importantly, these functions are not independent modules but are tightly coupled, such that perturbations along one axis propagate across the others. This systems-level perspective provides a coherent explanation for why mutations in ubiquitously expressed lamins can produce shared nuclear abnormalities while generating sharply tissue-selective disease phenotypes.

A consistent lesson from laminopathy research is the inadequacy of single-axis explanations. Classical distinctions between mechanical fragility and gene regulation defects remain conceptually useful, yet accumulating evidence indicates that these dimensions are mechanistically inseparable. Lamin perturbations frequently alter nuclear mechanics and chromatin architecture in parallel, with reciprocal feedback influencing whether a given mutation is buffered or progresses toward pathology in a specific tissue context [6,12,15]. Tissue specificity therefore emerges not from a single dominant pathway, but from the intersection of mutation-specific molecular lesions with local mechanical load, metabolic state, and developmental history [14,15,119].

Rare progeroid syndromes, particularly HGPS, have functioned as informative experimental extremes that expose vulnerabilities in nuclear organization. Their value lies less in modeling physiological aging per se than in clarifying how defects in lamin processing and nuclear envelope integrity propagate across molecular, cellular, and tissue scales. Studies of HGPS have demonstrated that chronic lamin processing defects can destabilize nuclear architecture, compromise stress tolerance, and accelerate tissue degeneration, while also highlighting the limits of extrapolating from severe, early-onset laminopathies to normative aging trajectories [44,46].

Notably, chromatin tethering and three-dimensional genome organization remain among the most consequential yet methodologically challenging aspects of lamin biology. LAD frameworks describe how lamins anchor heterochromatin and shape gene repression programs during differentiation [6,9]. However, LAD biology is heterogeneous, encompassing constitutive and variable LADs. It is further complicated by the ensemble -single-cell discrepancy, whereby population-averaged sequencing obscures cell-to-cell variability in peripheral chromatin positioning [9]. Disease-oriented multi-omics analyses extend this concept to progeroid contexts, revealing hierarchical chromatin disorganization—including altered LADs and TADs—linked to dysregulation of geroprotective and senescence-associated pathways, while remaining constrained by limited patient material and the in vitro nature of iPSC differentiation [61]. Recent mapping approaches that partition heterochromatin associations between lamin B1 and lamin B2 further suggest that closely related B-type lamins occupy distinct chromatin subcompartments, implying that genotype–phenotype relationships may depend on chromatin partitioning logic not captured by coarse A-type versus B-type distinctions [53].

Genome integrity has become an increasingly prominent lens for interpreting lamin function, particularly as the field connects nuclear mechanics to the choice of DNA repair pathways and chromosomal stability. Evidence demonstrates that lamin A/C scaffolds DNA repair factors and that mutant lamins can impair damage responses [11,43]. Meanwhile, unresolved questions remain regarding whether instability primarily arises from rupture-mediated DNA exposure or defective recruitment of repair machinery. Mechanistic studies identifying nucleoskeleton networks linking lamin A/C to core end-joining components—such as IFFO1/2 binding XRCC4 to promote NHEJ and restrain chromosome translocations—reinforce the view that nuclear lamina integrity contributes to both physical and biochemical microenvironments of DNA repair [42]. Complementary work indicates that nuclear deformability itself can influence repair outcomes and therapeutic sensitivities, further positioning lamins as regulators rather than passive bystanders in genome maintenance under stress [37,42].

A recurring implication is that lamin dysfunction frequently converges on aging-associated phenotypes, raising the question of whether lamin may serve as markers or drivers of biological aging. Lamin defects can promote chronic nuclear stress and contribute to senescence-associated inflammatory remodeling, yet senescent states remain heterogeneous and lack universal biomarkers [112,115]. Evidence that rare progerin-expressing or lamin-compromised cells exert disproportionate paracrine effects suggests a threshold-like mechanism, whereby low-frequency nuclear defects scale up to tissue-level pathology [147]. The identification of recurrent somatic progeroid mutations and clonal expansion of mutant vascular smooth muscle cells in human arteries extends this concept to vascular aging, while leaving open fundamental questions regarding causality and mutation induction [150].

The overarching outlook is therefore integrative and mechanistically disciplined. Laminopathies are best understood as disorders of nuclear system coordination, in which mechanical resilience, chromatin organization, genome maintenance, and stress-response wiring co-determine cell fate and tissue degeneration. The field now possesses conceptual frameworks and a growing set of structural and functional tools to move from correlation to causation. Yet, many clinically relevant questions—particularly tissue specificity and durable, safe therapy—require experimental designs that explicitly span scales from molecular interfaces to organ-level mechanics. Progress will likely come from combining high-resolution structural biology of lamin–chromatin contacts [20,48], quantitative nuclear mechanics in physiologically relevant microenvironments [8,26], lineage-aware human studies that can resolve mosaicism and clonal expansion [150], and carefully matched therapeutic strategies that acknowledge pathway pleiotropy and long-term risk [21,98]. Within this integrated framework, the long-standing paradox of tissue-selective disease from ubiquitous proteins becomes tractable. The goal is no longer to choose between mechanics and gene regulation, but to define how particular mutations reshape the coupled nuclear system under the specific constraints of each tissue.

## Figures and Tables

**Table 1:** Mutations in *LMNA*, *LMNB1*, and *LMNB2* and Their Associated Disease Phenotypes.

Gene	Mutation/Variant	Disease/Syndrome	Phenotype/Pathogenic Mechanism	References
*LMNA*	De novo *LMNA* point mutation c.1827C>T (p.G609G)	HGPS	Activates a cryptic splice donor site in exon 11; produces progerin (Δ50 aa, permanently farnesylated); nuclear envelope budding, lamina thickening, peripheral heterochromatin loss, impaired DNA repair, premature aging, cardiovascular disease	[22,78,79,80,81]
	p.E262K	Atypical/progeroid laminopathy	Links nuclear proteostasis imbalance to laminopathy-associated premature aging	[82]
	Other rare *LMNA* variants causing HGPS	HGPS (atypical)	Alternative *LMNA* mutations producing HGPS-like features	[82,83]
	Various *LMNA* mutations	Restrictive dermopathy	Tight translucent skin, joint contractures, pulmonary hypoplasia, neonatal lethality; disrupted lamin A production and nuclear envelope integrity	[84,85]
	p.R527H (homozygous)	MAD	Skeletal dysplasia, partial lipodystrophy, milder progeroid features; hierarchical chromatin disorganization, altered LADs, and shortened TADs	[61,62,86]
	p.L648R (mouse model)	Craniosynostosis-like/craniofacial deformity	Blocks prelamin A maturation; abnormal suture fusion, low bone density; disrupts bone stem-cell stemness and osteogenic differentiation	[87]
	Heterozygous *LMNA* variants	Atypical Werner syndrome	Progeroid phenotype overlapping with classical WRN-mutant Werner syndrome; phenotypic convergence between distinct progeroid mechanisms	[88]
	p.Q353R	DCM	Aberrantly traps TEAD1 at the nuclear periphery; impairs cardiac maturation gene expression; also sequesters VDR, blunting DNA repair gene expression (rescued by vitamin D in vitro)	[33,64]
	p.E161K	DCM	Large-scale destruction of the peripheral lamina; aberrant heterochromatin and intranuclear aggregates; disrupts SON binding and nuclear-genome organization	[60,89]
	p.K97E	DCM	Disrupts the peripheral lamina; leads to heterochromatin disorganization and intranuclear aggregate formation	[89]
	p.H222P (mouse *Lmna* ^H222P/H222P^)	DCM with conduction defects; EDMD-like striated muscle disease	Constitutive MAPK/ERK hyperactivation drives fibrosis and apoptosis in cardiomyocytes	[63,90]
	*LMNA* missense/loss-of-function	EDMD, LGMD1B	Early contractures, humero-peroneal weakness, conduction defects; late-onset proximal weakness; recurrent nuclear envelope rupture, DNA damage, and p53-mediated apoptosis under mechanical stress	[22,34,77,91,92]
	p. R249W	CMD	Early loss of walking ability; severe congenital striated-muscle phenotype	[93]
	p.R249Q (corrected by ABE in mice)	*LMNA*-related CMD/cardiac disease (mouse)	Adenine base editing precisely corrected R249Q and rescued the pathological phenotype, prolonging lifespan	[94]
	p.L35P (corrected by CBE in mice; modeled in zebrafish)	Muscular laminopathy/cardiac disease	CBE corrected L35P and rescued the phenotype in mice; in the *lmna* L35P zebrafish, muscle endurance was rescued by L-carnitine treatment	[94,95]
	p.R453W (zebrafish model)	Muscular laminopathy	Reduced muscle endurance; rescued by creatine treatment via activation of AMPK and mTOR pathways, improving swimming speed	[95]
	Intron 8 donor splice site variants	Diverse laminopathy phenotypes	Variable phenotypic expression; basis of clinical heterogeneity in laminopathies	[96]
	Splice variants near intron 11 (mouse *Lmna* c.1827C>T)	HGPS (mouse model)	Orthologous to the human HGPS mutation; growth retardation, vascular smooth muscle cell loss, and shortened lifespan	[97,98,99]
	p.R482W/R482Q/R482L	FPLD2	Heterozygous missense mutations in Ig-like fold; sequester SREBP1 at the nuclear periphery, impairing adipogenesis; loss of subcutaneous fat, insulin resistance, diabetes, dyslipidemia, hepatic steatosis	[100,101,102,103,104,105]
	p.K486N/K486/H506 residues	FPLD2	Mutations disrupt chromatin interactions and adipose-specific gene networks	[20,48,100]
	Novel/ultrarare heterozygous missense *LMNA* variants	FPLD2	Newly identified missense variants causing FPLD2; expand the mutation landscape beyond the R482 hotspot	[104]
	*LMNA* mutation (pediatric case)	FPLD2	Pediatric-onset FPLD2 managed with liraglutide	[103]
	Missense and non-missense *LMNA* variants	*LMNA* Cardiomyopathy	Variant location and type influence cardiovascular prognosis; missense and non-missense variants confer distinct outcomes	[106,107]
*LMNB1*	Non-coding *LMNB1* duplication (double overexpression)	ADLD	Lamin B1 overexpression; reduces LIF secretion; oligodendrocyte loss and demyelination; nuclear envelope invaginations and oxidative stress	[108,109,110,111]
	Downregulation *LMNB1* expression (non-point mutation)	Cellular senescence and physiological aging	Loss of peripheral heterochromatin, decreased H3K9me3, increased nuclear fragility, SASP activation; biomarker and contributor to aging	[57,110,112,113,114,115]
	Decreased *LMNB1* expression (*Fmr1^ΔExon^ ^8^* rat hippocampus)	Fragile-X-like nuclear morphology defects	Hippocampal nuclear abnormalities and glial alterations associated with *LMNB1* dysregulation	[116]
	*LMNB1* dysregulation (overexpression in cancers)	Cancer (context-dependent)	Altered expression in colorectal, prostate, and other malignancies; impacts nuclear stability and proliferation programs	[110,117]
*LMNB2*	p.R215Q	APL	Susceptibility variant; proposed to alter nuclear mechanics or chromatin interactions in preadipocytes, impairing adipocyte differentiation/survival	[117,118]
	p.A407T	APL	Heterozygous susceptibility variant predisposing to adipose tissue loss	[117,118]
	*LMNB2* dysregulation (over- or underexpression)	Cancer (multiple types: colorectal, prostate, others)	Context-dependent role; supports mitotic spindle assembly and chromosome segregation; thus, loss promotes mitotic errors and genomic instability; essential for DNA replication	[24,53,69,110,117]

Abbreviations: HGPS, Hutchinson-Gilford progeria syndrome; MAD, Mandibuloacral dysplasia; LAD, lamina-associated domains; TAD, topologically associating domains; DCM, Dilated cardiomyopathy; VDR, vitamin D receptor; TEAD1, TEA domain transcription factor 1; EDMD, Emery–Dreifuss muscular dystrophy; LGMD1B, Limb-girdle muscular dystrophy 1B; CMD, Congenital muscular dystrophies; CBE, cytosine base editing; ABE, adenine base editing; FPLD2, Dunnigan-type familial partial lipodystrophy; SREBP1, Sterol regulatory element-binding protein 1; ADLD, Adult-onset autosomal dominant leukodystrophy; LIF, Leukemia inhibitory factor; SASP, Senescence-associated secretory phenotype; APL, Acquired partial lipodystrophy.

## Data Availability

No new data were created or analyzed in this study. Data sharing is not applicable to this article.
